# Thoracoscopic management of easily neglected extralobar pulmonary sequestration presenting with torsion and infarction in children: three case reports and literature review

**DOI:** 10.3389/fped.2026.1815761

**Published:** 2026-07-02

**Authors:** Xiaolong Chen, Li Shen, Haifa Hong

**Affiliations:** Department of Cardiothoracic Surgery, School of Medicine, Shanghai Children’s Hospital, Shanghai Jiao Tong University, Shanghai, China

**Keywords:** abdominal pain, chest pain, children, extralobar pulmonary sequestration, infarction, thoracoscopic, torsion

## Abstract

**Background:**

Extralobar pulmonary sequestration (ELS) is a rare congenital pulmonary malformation, and reports of ELS complicated by torsion and infarction are exceedingly uncommon. Here, we report three pediatric cases of ELS complicated by torsion and infarction, aiming to enhance clinical awareness and understanding of this condition.

**Methods:**

Using the keywords “extralobar pulmonary sequestration, torsion, infarction, thoracoscopic, chest pain, abdominal pain, children”, we conducted a comprehensive literature review of all studies related to ELS with torsion in children from database inception to December 2025. We identified 16 articles describing ELS with torsion, comprising a total of 24 reported pediatric cases of pulmonary sequestration complicated by torsion.

**Results:**

Between May 2022 and November 2025, three children with ELS complicated by torsion and infarction were treated at Shanghai Children's Hospital. Two patients presented primarily with abdominal pain and chest pain, while one patient presented with abdominal pain alone. Contrast-enhanced chest computed tomography (CT) was performed in all cases: two patients were suspected of having intrathoracic soft-tissue masses, and one patient was diagnosed with pulmonary sequestration. All patients subsequently underwent thoracoscopic resection of the pulmonary sequestration. Postoperative pathological examination confirmed pulmonary sequestration with hemorrhagic necrosis in all three cases.

**Conclusions:**

Pulmonary sequestration with torsion should be considered in the differential diagnosis of patients presenting with chest/abdominal pain accompanied by an intrathoracic soft-tissue mass. With the continuous advancement of minimally invasive techniques, thoracoscopic surgery offers a favorable prognosis for pediatric patients with pulmonary sequestration complicated by torsion and infarction.

## 1 Introduction

Pulmonary sequestration (PS) is defined as a segment of nonfunctioning lung tissue that lacks an identifiable communication with the tracheobronchial tree and derives its arterial blood supply from one or more anomalous systemic arteries (1). PS is frequently misdiagnosed as lung cancer, pulmonary cystic lesions, or mediastinal tumors. Based on pleural investment, PS is classified into two subtypes: extralobar pulmonary sequestration (ELS), which is separated from adjacent normal lung by its own visceral pleura, and intralobar pulmonary sequestration (ILS), which is contained within the visceral pleura of the normal lung (2). Most ELS lesions are located between the lower lobe and the diaphragm (3–5). The feeding artery most commonly arises from the thoracic aorta, abdominal aorta, or other systemic vessels, whereas venous drainage typically returns to the right atrium via the azygos vein, hemiazygos vein, or the vena cava (6). In recent years, with the widespread implementation of antenatal ultrasonographic screening, the reported incidence of PS has increased (7). Most patients with ELS are asymptomatic and are therefore identified incidentally during routine prenatal screening or postnatal physical examinations. Nevertheless, in rare cases, ELS may undergo torsion, which can present with acute abdominal or chest pain.

## 2 Materials and methods

A systematic search was conducted across PubMed, Embase, Web of Science, and the Cochrane Library from inception to December 2025. The search strategy employed Boolean operators combining terms related to “extralobar pulmonary sequestration” “torsion,” and “infarction”. To ensure comprehensiveness, the reference lists of the retrieved articles were manually screened for additional relevant cases.

Eligibility criteria studies were included if they met the following criteria: (1) patients aged < 18 years; (2) a confirmed diagnosis of extralobar pulmonary sequestration via histopathology or a combination of surgical findings and cross-sectional imaging (CTA/MRI); and (3) documented torsion of the vascular pedicle resulting in tissue infarction. Exclusion criteria were: (1) adult patients (age>18 years); (2) cases of intralobar sequestration; and (3) studies with insufficient clinical data or those published only as abstracts. Titles and abstracts were screened by two independent reviewers, followed by a full-text evaluation. Discrepancies were resolved through consensus with a third senior clinician. To prevent double-counting, we meticulously cross-referenced institutional affiliations, patient demographics, and surgical dates across all reports. In instances where the same patient was described in multiple publications, only the most comprehensive report was included in the final analysis.

We identified 16 articles describing ELS with torsion (Table 1), comprising a total of 24 reported pediatric cases of pulmonary sequestration complicated by torsion. The cohort comprised 9 females and 15 males, with ages ranging from 3 to 15 years (median age: 10 years). The majority of cases were concentrated within the 10–13-year age bracket, and the youngest patient was 3 years old. Among these 24 patients, only 7 presented with chest pain, whereas abdominal pain was the most common symptom. All patients underwent preoperative imaging with non-contrast and/or contrast-enhanced CT or MRI; however, a suspected systemic feeding artery was visualized in only 3 cases. Pleural effusion was documented in all patients. Reported preoperative diagnoses included diaphragmatic hernia, tumor, pulmonary consolidation, ELS, and an indeterminate mass. Regarding the vascular anatomy, approximately 85% of the aberrant feeding arteries originated directly from the thoracic aorta, with a minority arising from the abdominal aorta or intercostal arteries. Following surgical resection—predominantly via video-assisted thoracoscopic surgery (VATS)—100% of the patients experienced immediate relief of acute abdominal or chest pain. Long-term follow-up demonstrated no postoperative complications or recurrence; all patients exhibited satisfactory physical development and preserved pulmonary function.

**Table 1:** Summary of extralobar pulmonary sequestration with torsion reported in literature.

Authors	Case series	Published year	Age, yrs	Sex	Main symptom	Location	Main imaging tool for diagnosis	Preoperative Diagnosis	Feeding artery On image
Huang et al. (8)	1	2004	13	Female	Abdominal pain and vomiting	Left	CT and MRI	Chest mass	Invisible
Shah et al. (9)	1	2010	11	Female	Abdominal pain and chest pain	Left	Contrast-enhanced CT	Chest mass	Invisible
Shah et al. (10)	1	2010	11	Female	Abdominal pain	Left	CT and MRI	Chest mass	Invisible
Uchida et al. (11)	1	2010	4	Male	Abdominal pain	Left	CT	Chest mass	Invisible
Chen et al. (12)	1	2011	13	Male	Abdominal pain	Left	CT and PET	Posterior mediastinal mass	Invisible
Kirkendall et al. (13)	1	2012	13	Male	Abdominal pain and chest pain	Left	CT and PET	Chest tumor	Invisible
Gawlitza et al. (14)	1	2013	11	Male	Abdominal pain and vomiting	Left	Contrast-enhanced CT and MRI	Chest mass	Invisible
Zucker et al. (4)	1	2013	6	Male	Abdominal pain and chest pain	Left	contrast-enhanced CT	Chest mass	Invisible
Choe et al. (15)	1	2015	10	Male	Abdominal pain	Left	Contrast-enhanced CT and MRI	ELS with hemorrhagic infarction	Visible
Ah Son et al. (16)	1	2019	13	Female	Abdominal pain and chest pain	Left	CT	ELS	Invisible
Yokota et al. (17)	1	2019	15	Male	Abdominal pain	Right	CT and MRI	ELS with infarction	Invisible
Yang et al. (5)	1	2020	10	Male	Chest pain	Left	Contrast-enhanced CT	Diaphragmatic hernia	Visible
Walcutt et al. (3)	1	2021	13	Male	Abdominal pain	Left	CT and MRI	ELS with infarction and hemorrhage	Invisible
Ti et al. (18)	6	2022	3–12	Three Males, Three Females	Abdominal pain/chest pain/abdominal distension and vomiting	Two Left, Four Right	Contrast-enhanced CT and Color Doppler ultrasound	Neurogenic tumor/ELS/Lung onsolidation	One Visible, Five Invisible
Wu et al. (19)	1	2023	11	Male	Abdominal and chest pain	Left	Contrast-enhanced CT	Posterior mediastinal mass	Invisible
Jiang et al. (20)	4	2024	3–10	Two Males,Two Females	Abdominal pain	Not mentioned	CT	Intrathoracic soft tissue mass	Invisible

## 3 Results

### 3.1 Case presentation

#### 3.1.1 Patient 1

**Figure 1:**
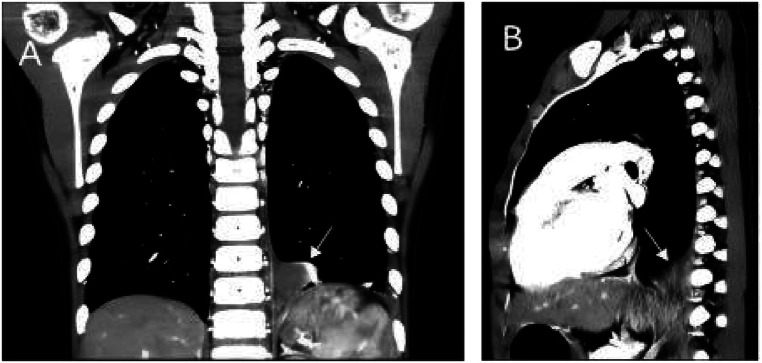
Contrast-enhanced chest CT. **(A)** A soft-tissue mass (arrow) is noted with peripheral enhancement along its margins. **(B)** A soft-tissue mass (arrow) is shown with no definite arterial feeding vessel identified.

**Figure 2:**
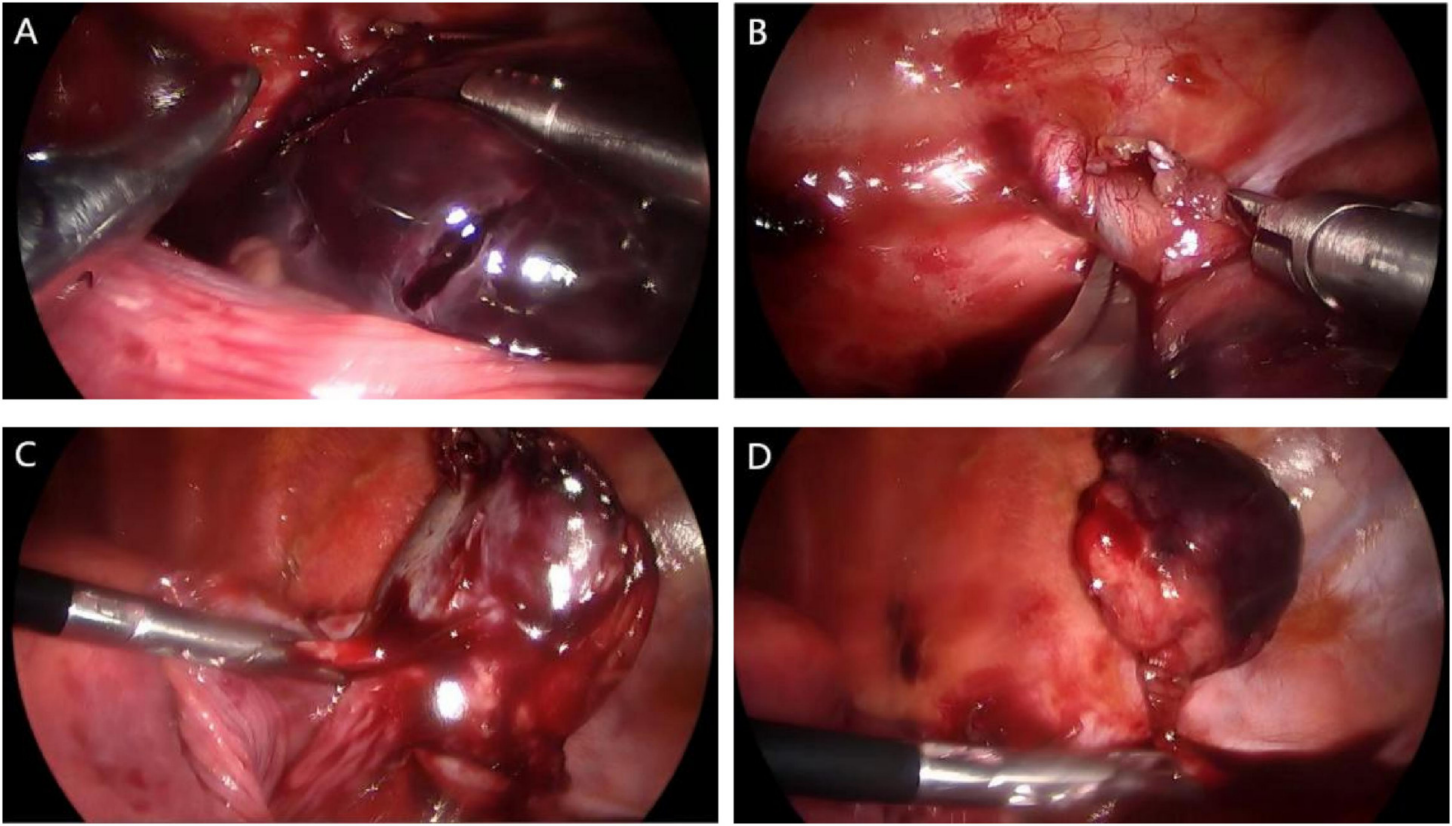
Intraoperative thoracoscopic view. **(A)** Infarcted sequestered lung tissue with hemorrhagic pleural effusion. **(B)** Torsion of the basal vascular pedicle. **(C)** Adhesion of the sequestered lung tissue to the adjacent lobe. **(D)** An intact sequestration morphology.

A 7-year-old girl was admitted with a 3-day history of abdominal pain and a 2-day history of chest pain. On presentation, her main complaints were abdominal and chest pain. She had been previously healthy, with no history of recurrent respiratory tract infections. She had received bacille Calmette–Guérin (BCG) vaccination and denied any history of tuberculosis exposure. In addition, there was no history of ingestion of contaminated or raw/cold food. Her personal and family histories were unremarkable. Physical examination revealed decreased breath sounds at the left lung base, while the remainder of the examination was normal. Laboratory testing showed leukocytosis (white blood cell count, 17.85 × 10^9/L) with neutrophilia (68.9%); point-of-care C-reactive protein (CRP) was ≤5 mg/L, and the eosinophil count was within the normal range. Tumor markers (carbohydrate antigens, CEA, AFP, NSE, HCG, and urinary VMA) were all within normal limits. No significant abnormalities were observed in blood biochemistry or coagulation parameters. Contrast-enhanced chest CT demonstrated a left paravertebral soft-tissue mass located superior to the diaphragm with associated pleural effusion. The lesion showed mild peripheral enhancement, and no aberrant systemic feeding artery was identified (Figure 1). Abdominal imaging revealed no evidence of an intra-abdominal mass. Thoracoscopic resection of the lesion was performed. Intraoperatively, a dark purple infarcted mass with hemorrhagic pleural effusion was noted. The feeding artery originated from the celiac trunk, with torsion of the pedicle, and the lesion exhibited inflammatory adhesions to the adjacent left lower lobe (Figure 2). After adhesiolysis between the sequestered lung tissue and the left lower lobe/diaphragm, the pedicle was occluded with absorbable vascular clips, ligated with sutures, and divided using an ultrasonic scalpel; a chest drain was placed. Postoperative histopathology confirmed pulmonary sequestration with infarction. Histopathological examination revealed extensive areas of hemorrhagic necrosis characterized by numerous irregular, anastomosing, thick-walled blood vessels. At the margins, focal residual pulmonary parenchyma and fibroadipose tissue were observed. The interstitium exhibited significant infiltration of both acute and chronic inflammatory cells, accompanied by fibroblastic proliferation and histiocytic aggregates. Additionally, evidence of partial bronchial hyperplasia was noted. The patient's pain improved after surgery, and no discomfort was reported during follow-up after discharge. To date, the patient has undergone continuous postoperative follow-up for three years, during which her physical development and pulmonary function have remained within normal limits. A follow-up chest CT scan performed one year after the procedure demonstrated normal bilateral lung development with no evidence of inflammatory changes or lesions.

#### 3.1.2 Patient 2

**Figure 3:**
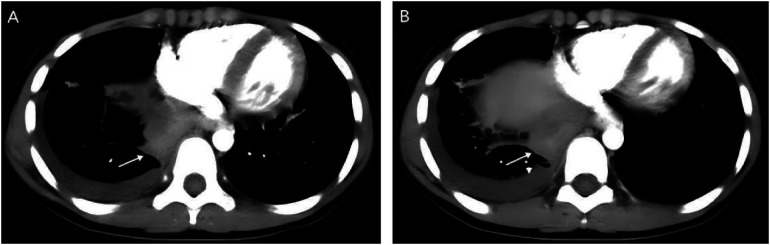
Contrast-enhanced chest CT. **(A)** A soft-tissue mass (arrow) with ill-defined margins, adherent to the adjacent lung parenchyma. **(B)** A soft-tissue mass (arrow) adherent to the diaphragm with associated pleural effusion.

**Figure 4:**
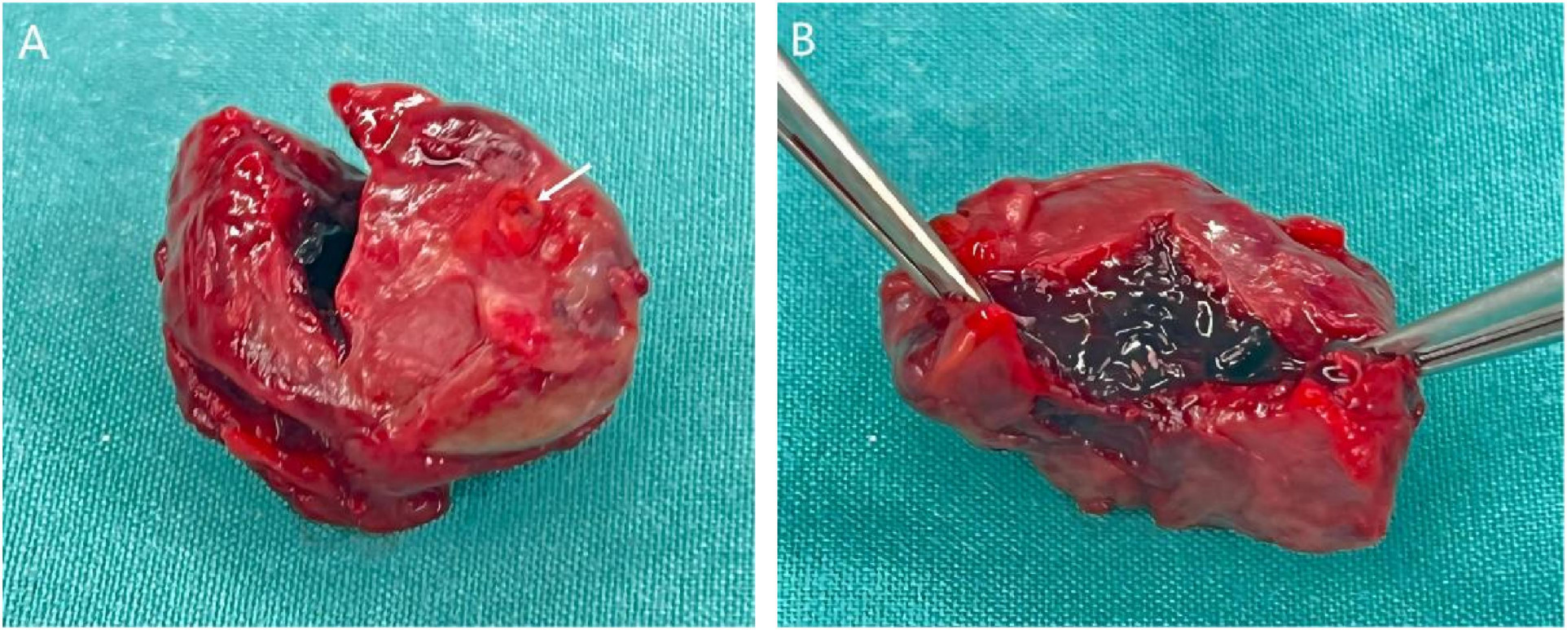
Gross appearance of the resected pulmonary sequestration. **(A)** Ovoid, soft-textured tissue; dark red surface of the mass. The arrow indicates the feeding artery. **(B)** Cut section shows internal old clots.

**Figure 5:**
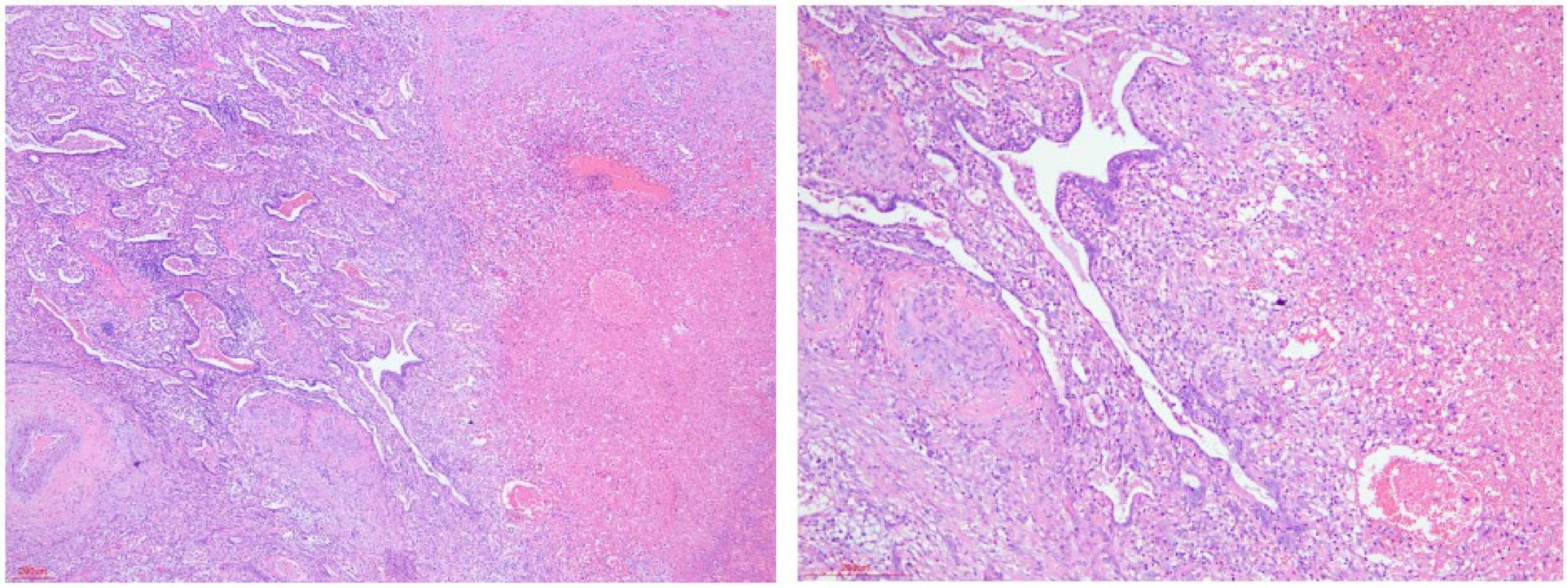
Histologically, extensive hemorrhagic necrosis and proliferative dilation of the bronchial structures were observed.

A 10-year-old boy was admitted with a 7-day history of abdominal pain. On presentation, his main symptoms were abdominal pain accompanied by vomiting. He had been previously healthy and had no history of recurrent respiratory tract infections. He had received BCG vaccination and denied any history of tuberculosis exposure. In addition, there was no history of ingestion of contaminated or raw/cold food. His personal and family histories were unremarkable. Physical examination revealed decreased breath sounds and reduced tactile vocal fremitus at the right lung base; the remainder of the examination was normal. Complete blood count results showed that the white blood cell count, neutrophil percentage, and eosinophil count were within normal ranges. Carbohydrate antigen 19-9 (CA19-9) was elevated to 118.09 U/mL, whereas other tumor markers (CEA, AFP, NSE, HCG, and urinary VMA) were within normal limits. No significant abnormalities were observed in blood biochemistry or coagulation parameters. Contrast-enhanced chest CT revealed a right paravertebral soft-tissue mass located superior to the diaphragm with associated pleural effusion. The mass showed no obvious enhancement, and no aberrant systemic feeding artery was identified (Figure 3). Abdominal imaging demonstrated no evidence of an intra-abdominal mass. Given that malignancy could not be excluded, thoracoscopic resection of the lesion was performed. Intraoperatively, a dark red necrotic mass with a large amount of hemorrhagic pleural effusion was observed. The feeding artery arose from the descending aorta, with torsion of the pedicle, and the lesion exhibited inflammatory adhesions to the adjacent lower lobe. After adhesiolysis between the sequestered lung tissue and the right lower lobe/diaphragm, the feeding artery was ligated with Hem-o-lok clips and the sequestered lung tissue was resected (Figure 4). Postoperative histopathology confirmed pulmonary sequestration with infarction. Microscopic examination revealed extensive areas of hemorrhagic necrosis and proliferative fibrous tissue. Remnants of irregular, thick-walled vessels were discernible within the background. Locally, hyperplastic and dilated bronchial structures were observed, lined by pseudostratified ciliated columnar epithelium or cuboidal epithelium (Figure 5). The patient's pain improved after surgery, and no discomfort was reported during follow-up after discharge. To date, the patient has been followed for 10 months postoperatively, during which he has exhibited age-appropriate growth and development. Follow-up laboratory testing at three months post-resection showed that serum CA19-9 levels had normalized. Additionally, chest radiography performed six months after the procedure was unremarkable.

#### 3.1.3 Patient 3

**Figure 6:**
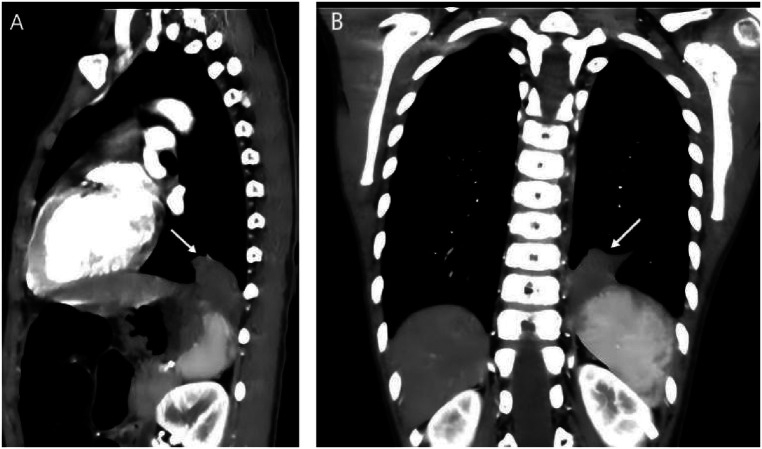
Contrast-enhanced chest CT. **(A)** A soft-tissue mass (arrow) is noted with well-defined margins. **(B)** A soft-tissue mass (arrow) is shown with no definite arterial feeding vessel identified.

**Figure 7:**
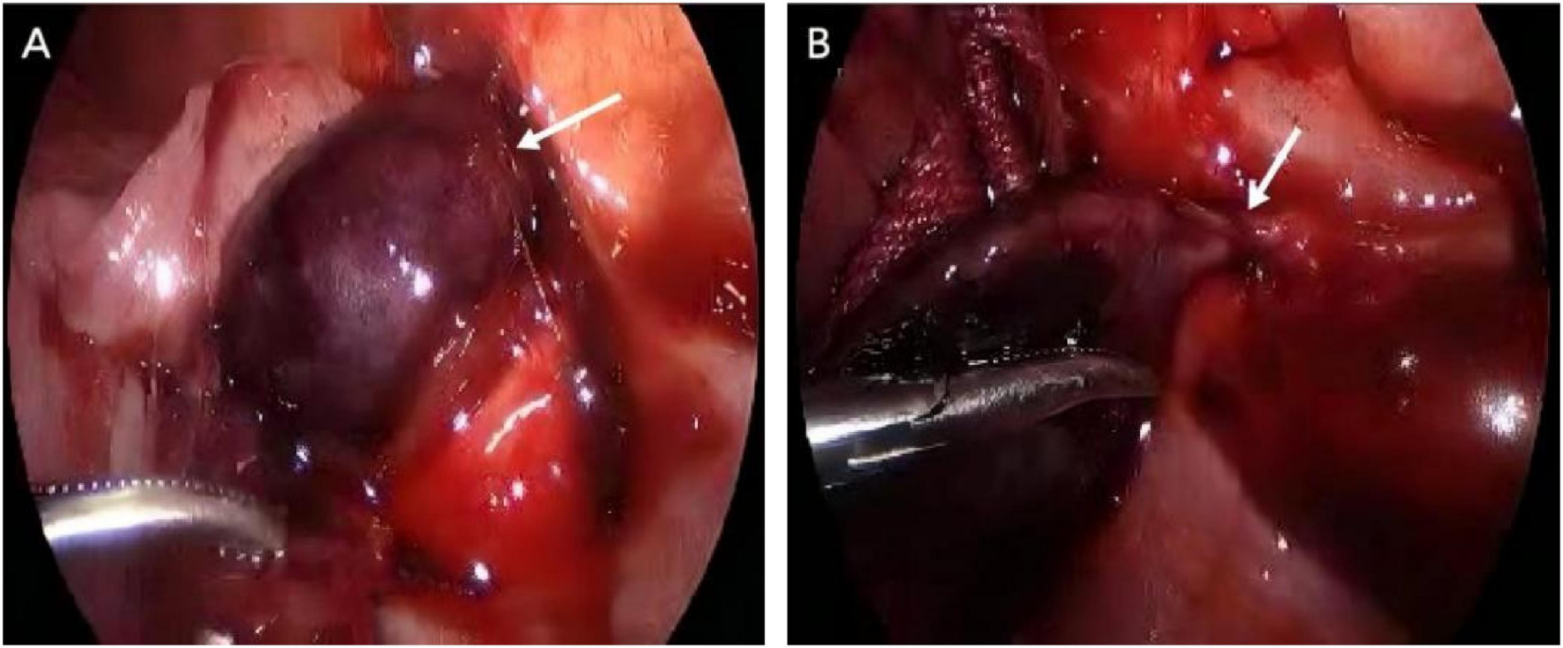
Intraoperative thoracoscopic view. **(A)** Infarcted sequestered lung tissue (arrow), adherent to the adjacent lobe, chest wall, and diaphragm. **(B)** The arrow indicates the basal feeding artery.

A 6-year-old boy was admitted with a 2-day history of abdominal pain and a 1-day history of chest pain. On presentation, his predominant symptoms were abdominal pain accompanied by vomiting. He had been previously healthy, with no history of recurrent respiratory tract infections. He had received BCG vaccination and denied any history of tuberculosis exposure. In addition, there was no history of ingestion of contaminated or raw/cold food. His personal and family histories were unremarkable. Physical examination was unremarkable. Laboratory testing showed a neutrophil proportion of 75.4%, while the total white blood cell count and eosinophil count were within normal ranges. Tumor markers (carbohydrate antigens, CEA, AFP, NSE, HCG, and urinary VMA) were within normal limits. No significant abnormalities were observed in blood biochemistry or coagulation parameters. Contrast-enhanced chest CT demonstrated a left paravertebral soft-tissue mass located superior to the diaphragm with associated pleural effusion. The lesion showed no obvious enhancement, and no aberrant systemic feeding artery was identified (Figure 6). Abdominal imaging revealed no evidence of an intra-abdominal mass. Based on our prior diagnostic and therapeutic experience, the patient presented with characteristic abdominal pain concomitant with chest pain; intra-abdominal pathologies were effectively excluded. Contrast-enhanced chest CT demonstrated a well-circumscribed paraspinal soft-tissue lesion, morphologically suggestive of ELS. Notably, the lesion exhibited minimal to no enhancement during the contrast-enhanced phase compared to the adjacent normal lung parenchyma or the contralateral side, and no aberrant systemic feeding artery was identified, accompanied by localized pleural effusion, so pulmonary sequestration with pedicle torsion was suspected; therefore, thoracoscopic resection of the sequestered lung was performed as planned. Intraoperatively, a grayish-brown infarcted mass with a large amount of hemorrhagic pleural effusion was observed (Figure 7). A systemic feeding artery arising from the descending aorta was identified and ligated using Hem-o-lok clips, and a chest drain was placed. Postoperative histopathology confirmed pulmonary sequestration with infarction. Microscopic evaluation revealed that a significant portion of the pulmonary parenchyma had undergone necrosis, accompanied by widespread hemorrhage and inflammatory cell infiltration. Interspersed within the necrotic areas, dilated and congested blood vessels with focal hemorrhagic changes were also identified. The patient's pain improved after surgery, and no discomfort was reported during follow-up after discharge. At the 4-month postoperative follow-up, the patient's physical development remained within normal limits, with no reported clinical discomfort.

The three pediatric cases (aged 6–10 years) shared several distinct clinical and radiological hallmarks. Clinically, all patients presented with acute, predominant abdominal pain, frequently accompanied by chest pain or vomiting, despite having no prior history of respiratory infections. Physical examination typically revealed diminished breath sounds at the affected lung base. Laboratory findings were variable, ranging from leukocytosis and neutrophilia to a significant elevation of CA19-9 in one instance, while other tumor markers remained normal. Radiologically, contrast-enhanced CT consistently demonstrated a well-defined, supra-diaphragmatic paravertebral soft-tissue mass with associated pleural effusion. A critical common feature was the minimal to no enhancement of the lesion and the non-visualization of an aberrant systemic feeding artery on preoperative imaging. Surgical intervention in all cases confirmed the diagnosis of extralobar pulmonary sequestration with pedicle torsion, manifesting as a necrotic, infarcted mass (ranging from dark purple to grayish-brown) accompanied by hemorrhagic pleural effusion. The feeding arteries originated from either the descending aorta or the celiac trunk. The structured summary of the three cases is showed in (Table 2). Comparative analysis with cases documented in the literature indicates a male predominance and a primary clinical presentation of abdominal pain. Furthermore, PS with torsion appears to favor the left side. Notably, while feeding arteries were identifiable in three cases reported in the literature, none were visualized on contrast-enhanced CT within our series. Given these diagnostic hurdles, preoperative radiological diagnosis remains challenging, with the majority of cases necessitating intraoperative findings and postoperative pathological confirmation for a definitive diagnosis.

**Table 2:** Structured summary of the three cases.

Case no.	Case 1	Case 2	Case 3
Age (years)	7	10	6
Sex	Female	Male	Male
Presenting symptoms	Abdominal pain and chest pain	Abdominal pain and vomiting	Abdominal pain and chest pain
Vital signs (temperature, heart rate, blood pressure, oxygen saturation)	37.3 ℃, 120 bpm, 119/76 mmHg, 99%	36.8 ℃, 90 bpm, 100/66 mmHg, 98%	38 ℃, 130 bpm, 115/76 mmHg, 100%
Side	Left	Right	Left
Lesion size	4*3*2 cm	3*2.5*2 cm	3.5*2.5*1.5 cm
Imaging features	A soft-tissue mass is noted with peripheral enhancement along its margins.	An ill-defined soft tissue mass adherent to the adjacent lung tissue, accompanied by moderate pleural effusion.	A soft-tissue mass is noted with well-defined margins.
Preoperative diagnosis	Chest mass	Chest mass	Pulmonary sequestration with torsion
Operative findings	Pulmonary sequestration with torsion infarction and pleural effusion	Pulmonary sequestration with torsion infarction and hemorrhagic pleural effusion	Pulmonary sequestration with torsion infarction and pleural effusion
The origin of the aberrant feeding arteries	Celiac trunk	Descending aorta	Descending aorta
Operative time (min)	85	100	55
Blood loss (mL)	20	50	5
Pathology	Pulmonary sequestration with hemorrhagic necrosis	Pulmonary sequestration with hemorrhagic necrosis	Pulmonary sequestration with hemorrhagic necrosis
Postoperative course	Favorable postoperative recovery	Favorable postoperative recovery	Favorable postoperative recovery
Length of hospital stay (days)	9	10	8
Follow-up duration (months)	36	10	4

The symbol “*” denotes multiplication (length × width × height).

## 4 Discussion

Pulmonary sequestration was first described by Huber in 1877 and was subsequently termed “sequestration” by Pryce in 1946 (21). It is characterized by dysplastic lung tissue that becomes isolated during embryogenesis, lacks normal communication with the tracheobronchial tree, and receives an aberrant systemic arterial supply (22).

Pulmonary sequestration is uncommon, with a reported incidence of 0.15%–1.7% (23). Extralobar sequestration (ELS) is less frequent than intralobar sequestration (ILS). Prior reports suggest a male predominance for ELS, with a male-to-female ratio of approximately 3:1 to 4:1 (24, 25). The data statistics showed that the three cases reported in this article account for 2.6% of the total number of ELS cases at our center during the same period (3/114). ELS is most often located on the left side, typically between the left lower lobe and the diaphragm.

The mechanisms and triggers of torsion in pulmonary sequestration remain poorly understood. Torsion may be facilitated by the absence of normal parenchymal attachment, which increases the mobility of ELS and its vascular pedicle. Physical activity or increased respiratory effort has been proposed as a precipitating factor; in some cases, symptom onset followed vigorous exercise (3).

The correlation between pulmonary sequestration torsion and markedly elevated CA19-9 levels can be elucidated through the “release-following-infarction” mechanism. Literature suggests that CA19-9 is not organ-specific and can be produced by the bronchial and alveolar epithelia (26). In PS, the dysplastic lung buds often lack a functional connection to the tracheobronchial tree, leading to the intracellular and interstitial accumulation of these antigens. When torsion occurs, the resulting acute ischemia and venous congestion lead to localized tissue hypoxia and cell death. We hypothesize that the destruction of the epithelial-blood barrier during infarction facilitates the leakage of accumulated CA19-9 into the bloodstream. Furthermore, the pro-inflammatory milieu induced by acute torsion may upregulate the expression of glycosyltransferases, potentially amplifying antigen production. Clinically, recognizing this phenomenon is crucial to avoid a misdiagnosis of malignancy. The rapid normalization of CA19-9 levels following surgical resection further substantiates that the elevation was a transient consequence of tissue injury rather than a malignant process.

ELS is frequently asymptomatic and may be detected incidentally. CTA and MRA are commonly used to identify the aberrant arterial supply and support diagnosis. Color Doppler ultrasound may also demonstrate feeding vessels, particularly when the lesion is adjacent to the diaphragm or liver, and is attractive for prenatal and postnatal assessment due to its noninvasiveness. In a retrospective Chinese cohort of 2,625 cases, arterial supply most commonly arose from the thoracic aorta (76.55%) and abdominal aorta (18.47%), while venous drainage predominantly returned to the pulmonary veins (90.97%) (27).

On contrast-enhanced CT, torsed ELS may present as a pleural-based mass with absent enhancement or peripheral-only enhancement, a nonvisualized or indistinct feeding artery, and pleural effusion. In our cases, minimal or rim enhancement was consistent with infarction.

Visualization of a systemic feeding artery strongly supports the diagnosis of ELS (28). However, torsion or thrombosis of the feeding vessel can cause flow stagnation and reduced perfusion, leading to nonvisualization of the feeding artery on imaging and making preoperative diagnosis challenging (28). This phenomenon is primarily driven by the mechanical kinking and luminal collapse of the vascular pedicle during the rotational event. Furthermore, the initial compression of low-pressure venous drainage leads to severe tissue congestion and an abrupt increase in retrograde resistance. This hemodynamic stagnation, coupled with potential secondary thrombosis triggered by endothelial injury, prevents the opacification of the feeding artery by the contrast agent, resulting in its disappearance on cross-sectional imaging. Among 24 reported cases, only three were correctly diagnosed before surgery. Even so, careful review of CT/MR images may still reveal suggestive features and the typical location of ELS, which can aid decision-making.

The predisposition of ELS to torsion is fundamentally linked to its anatomical independence and the morphometry of its vascular pedicle. Specifically, a high length-to-width ratio of the pedicle and a voluminous mass significantly elevate the rotational torque. Elongated vascular pedicles facilitate an increased rotational radius, effectively mimicking a “pendulum effect.” Consequently, slender pedicles exhibit a significantly higher propensity for axial rotation compared to shorter, more robust ones. Furthermore, voluminous lesions—typically exceeding 3–5 cm in diameter—possess a greater moment of inertia. This makes them more susceptible to gravity-induced rotation around the pedicle during postural shifts or diaphragmatic excursions. Additionally, ELS masses located freely near the mediastinum carry a markedly elevated risk of torsion due to the lack of extrinsic mechanical support, in contrast to lesions sequestered within pulmonary fissures or anchored along the paraspinal region. While ultrasound remains a valuable screening tool, contrast-enhanced CT and MRA exhibit superior diagnostic sensitivity by effectively visualizing the “whorl sign” and the abrupt termination of the aberrant artery, facilitating timely surgical intervention to prevent hemorrhagic gangrene.

In children, torsed ELS may initially manifest with abdominal pain, which can contribute to misdiagnosis or delayed diagnosis. Symptoms depend on lesion location and the child's ability to describe discomfort. When torsion occurs near the left diaphragmatic pleura, young children may report abdominal rather than chest pain, potentially due to diaphragmatic irritation and referred pain. Pleural effusion—often hemorrhagic—may result from obstruction of venous and lymphatic drainage caused by pedicle torsion. In this study, two cases were admitted to the general surgery or gastroenterology departments due to abdominal pain, with abdominal pain as the primary complaint for diagnosis and treatment. The experienced attending physician systematically investigated the cause of the illness in a short period and identified that the source of the pain was located in the chest. If the clinicians had not considered chest CT or ultrasound examination, the condition could have been delayed, and in severe cases, it might lead to severe infections or shock. Therefore, torsed ELS should be considered in pediatric patients presenting with abdominal pain accompanied by an intrathoracic mass, although this entity is rare.

Some studies suggest that ELS can remain asymptomatic or regress, supporting conservative management in selected cases (29). In our view, ELS still carries risks of infection and torsion. Large lesions may also exert a mass effect and compromise intrathoracic organ function in children with limited thoracic reserve. Our retrospective study revealed a 2.6% incidence of torsion-induced infarction in ELS. While numerically classified as a “low-frequency event,” this figure represents a clinically significant risk rather than a mere sporadic occurrence, especially when considering the inherent rarity of PS and the catastrophic nature of torsion. These findings prompt a critical reassessment of clinical management for asymptomatic patients: shifting from conservative “watchful waiting” toward proactive elective surgical intervention, guided by specific radiological characteristics. Video-assisted thoracoscopic surgery (VATS) has been increasingly applied for resection of torsed ELS. Given the diagnostic uncertainty in many cases, VATS provides both direct visualization and definitive treatment. All three patients in our institution successfully underwent VATS. Despite the presence of hemorrhagic pleural effusion and dense inflammatory adhesions, complete resection was achieved in all cases without conversion to open thoracotomy. Clinical follow-up, ranging from 4 to 36 months, demonstrated a 100% resolution rate with no recurrence or long-term complications. These outcomes are consistent with the favorable long-term prognosis reported in the 24-case literature review. The high-definition visualization afforded by VATS facilitates precise adhesiolysis, thereby minimizing the risk of iatrogenic injury to the adjacent normal lung parenchyma or the diaphragm. In the pediatric population, particularly school-aged children, this minimally invasive approach circumvents extensive thoracic scarring, reducing the potential psychological impact while preserving the integrity and function of the developing respiratory musculature. For lesions localized in the posterior mediastinum, paraspinal region, or superior to the diaphragm, the superior panoramic perspective of VATS offers significant advantages over traditional open surgery. Accordingly, we favor thoracoscopic resection as a practical option for ELS, particularly when complications are suspected. However, compared with open thoracotomy, the inherent friability and hemorrhagic tendency of infarcted tissues necessitate higher technical proficiency in endoscopic ligation and electrocoagulation-based hemostasis. Consequently, these factors may elevate the risk of major intraoperative hemorrhage and contribute to a more prolonged operative duration.

## 5 Conclusions

In conclusion, when a child presents with sudden-onset abdominal pain or chest pain, and CT reveals an intrathoracic soft-tissue density mass, clinicians should suspect torsion of ELS after excluding abdominal and genitourinary etiologies. As noted above, identification of a systemic feeding artery strongly supports the diagnosis of ELS; absence of this classic finding may indicate torsion. Given the risk of torsion-related infarction, early surgical intervention is recommended once ELS is diagnosed. Thoracoscopic resection for ELS is generally safe, is associated with minimal postoperative complications, and yields a favorable prognosis.

## Data Availability

The raw data supporting the conclusions of this article will be made available by the authors, without undue reservation.
